# Perception and experiences of sexual harassment among women working in hospitality workplaces of Bahir Dar city, Northwest Ethiopia: a qualitative study

**DOI:** 10.1186/s12889-021-11173-1

**Published:** 2021-06-11

**Authors:** Mulugeta Dile Worke, Zewdie Birhanu Koricha, Gurmesa Tura Debelew

**Affiliations:** 1 Department of Midwifery, College of Health Sciences, Debre Tabor University, Debre Tabor, Ethiopia; 2 Department of Health, Behavior, and Society, Faculty of Public Health, Jimma University, Jimma, Ethiopia; 3 Department of Population and Family Health, Faculty of Public Health, Jimma University, Jimma, Ethiopia

**Keywords:** Transactional sex, Factors, Effects, Hospitality, Ethiopia

## Abstract

**Background:**

Workplace sexual harassment is a public health problem that depends on gender, context, and perceived ideology. Although studies have documented the prevalence and consequences of workplace sexual harassment worldwide, victims’ perceptions and experiences are still poorly understood in low and middle-income countries, particularly Ethiopia. Female workers in the hospitality industry, including hotels, bars, restaurants, fast-food restaurants, and cafeterias, are particularly affected. Hence, this study aimed to explore sexual harassment perceptions and experiences among women working in these workplaces.

**Methods:**

An exploratory qualitative study was conducted from 1 January to 30 August 2019. Data were collected from female employees and key informants from several hospitality workplaces in Bahir Dar City. Data were collected through focus group discussions, in-depth interviews, and key-informant interviews. Women who experienced sexual harassment were selected using the snowball method, and key informants were recruited purposefully. Six focus group discussions, ten in-depth interviews, and thirteen key informant interviews were conducted. Data were analysed using the ATLAS ti version 8.4.24.

**Results:**

In this study, most participants perceived that sexual harassment is pressuring, threatening, touching, abducting sexual advances, and experiencing verbal, physical, and non-verbal types. Similarly, the perceived risk factors were related to the organisations, the customers, and the victims, with the consequences being work-related, health-related, financial-related, and family-related.

**Conclusions:**

Workplace sexual harassment in hospitality workplaces is poorly understood, but many women experience it. A variety of factors also caused it, and it influenced both organisations and people. Public awareness programs, pre-service preparation, in-service training, prevention, and psychosocial support are needed. Similarly, policies and strategies for the organisations should be developed and implemented.

**Supplementary Information:**

The online version contains supplementary material available at 10.1186/s12889-021-11173-1.

## Background

The world is looking better for women because of a decline in early marriage, increased involvement in leadership and politics, gender equality by reforming legislation, and 39% inclusion in the workforce [1]. However, despite their achievements, they continue to face challenges concerning sexual and reproductive health and rights. Workplace sexual violence (WSV) is one of the most serious sexual and reproductive health issues [2]. According to our systematic review and meta-analysis, workplace sexual harassment (WSH) is the most common form of WSV [3]. It has been viewed from legal, psychological, and public perspectives [4, 5]. It is defined objectively in the legal context while subjectively explaining it from a the psychological perspective [6]. This research focuses on the experiences of WSH victims and describes WSH from a psychological perspective. Accordingly, WSH includes unwelcome verbal, non-verbal, or physical sex-related conduct that the recipient views as offensive and has a detrimental effect on the victim’s well-being and work performance [7, 8].

The persistence and pervasiveness of workplace sexual harassment and its implications in various workplaces have been  well documented in the literature [9–12]. Women are disproportionately affected by WSH due to their working status, the type of work they do, and the conditions in the field they work in [2]. This issue may also harm their safety, health, and well-being [2, 12]. Emotional, psychological, professional, and health-related effects can occur [12, 13], resulting in costs worldwide, especially in low and middle-income countries [14]. Thus, well-established social assets, including social networks and tailored reproductive health knowledge, are needed to decrease WSH vulnerability [15]. Consequently, the Sustainable Development Goals [1], United Nations women and the International Labour Organization acknowledged this issue. These organisations also called for fundamental reform to ensure that all women have safe, secure, and respectful work environments [2].

Nevertheless, given the increasing number of hospitality industries, more women enrolment than men, and the more precarious nature of the job [16], there is a concern about WSH prevalence and the severe consequences [2]. This is a global problem because of young and minor employees with income *instability, stress, and* dependence on supervisors, managers, and customers. On the other hand, those working in low and middle-income countries’ hospitality industries are particularly unorganised and vulnerable [17]. Similarly, because of the differences in understanding, experience, perceived risks, and implications based on factors such as gender, background, and perceiver ideology, the WSH is still a debatable and unsettled problem worldwide, especially in low and middle-income countries [12, 18].

As a result, studies have reported that WSH is a severe public health concern that affects 42% of women working in hospitality jobs in the United States [19], 74.6% in 27 European countries [20], 89% in Australia [21], 50% in the Nordic Region [22], and 60% in Taiwan [23]. It is also a significant public health issue in Sub-Saharan African hospitality workplaces, such as Accra, Ghana [11, 24] (49.4%), Cameroon (98.8%) [25], Zimbabwe (78%) [8], and South Africa (14%) [26].

Precarious jobs [27–30], sexually objectified environment [31, 32], tolerance of sexual harassment [33–35], psychosocial safety climate [36–43], and complaint procedures have also been related to WSH [16–18]. In China, studies have revealed that tradition [44] and abusive supervision are linked to service performance [45] and WSH. Employees’ socio-economic status [46], workplace culture [31], unmet expectations of employees, inefficient organisational management, inappropriate professional communication, factors related to employees [47], and customers, supervisors, and co-workers [48] have all been established as predisposing factors for WSH. A recent review also summarised the causes as structural (e.g., causes related to the tourism sector structure and the nature of its employment), managerial, and widespread beliefs and norms in hospitality workplaces [17]. Previous research, on the other hand, had left out the employee and agent/broker considerations.

Furthermore, literature shows that [6, 17, 49] employees in various occupations are exposed to WSH from customers, co-workers, supervisors, and subordinates [50, 51]. These, in turn, affect organisations and each victim [5, 50] and are  widely viewed as a significant and prevalent problem, especially in occupations involving interpersonal contacts [49, 52]. There is still a gender, context, and ideology-based disparity in understanding, experience, and coping strategies in these occupations [18], and there are no validated measures to gain in-depth insights into hospitality WSH. As a result, recognising local perceptions, interactions, causes, and implications and valuing the status with validated resources helps meet international development agendas.

Despite immense pressure on WSH, advancements in policy development, and extensive research, WSH persists. Subsequently, the extent of the problem underlines the need for further research. However, the research on incidence rates can fail to accurately characterise the reality that confounds the WSH definition [18] owing to the lack of agreement on the WSH definition [4] and the misunderstanding of WSH terminologies. Some research has focused on the effects of WSH on mental and behavioural well-being, employment, and physical health. However, the reproductive health effects of WSH, such as transactional sex, commercial sex work, sexually transmitted infections (STIs), including HIV, and menstrual disorders, are rarely recognised and are not well understood. Consequently, this problem deters women’s capacity, which nearly mobilises half of the world’s business and endangers nearly all international Agendas’ attainability.

Likewise, in Ethiopia, although proclamation number 414/2004 prohibits WSH and prescribes simple imprisonment for the perpetrator [53] and is considered a prohibited act of workplace under proclamation number 1156/2019 [54], WSH in the hospitality workplace has been a secret issue until recently [3]. Only a few studies among commercial sex workers [55, 56], health care providers [57–59], restaurant workers [60], university students [61], female faculty and staff [62], and female civil servants [63] in limited areas have reported the level of workplace sexual violence. However, none of these studies considered people’s perceptions, experiences, and perceived risks of WSH. Moreover, although these concerns are essential for developing successful WSH prevention programs for women employed in the hospitality industry, most initiatives to mitigate reproductive health issues such as HIV/AIDS, unsafe abortion, and unintended pregnancy did not include WSH. Thus, this study aimed to explore women’s perceptions and experiences with WSH in the city administration’s hospitality workplaces in northwestern Ethiopia.

## Methods

### Study setting

This exploratory study was conducted in Bahir Dar city, Amhara national regional state capital, Ethiopia. Most hospitality workplaces are situated in the town, mainly because recreational centres are favourable for enjoyment. According to the Bahir Dar Special zone report in 2018/19, the Bahir Dar population is 356,757 (296, 532 urban, and 60,225 rural), of which 187,918 were female. It is one of the tourist destinations in this region. The number of people eating, drinking, and enjoying outside their homes is expected to increase, demanding more hotels, restaurants, and cafeterias. The estimated average size of female employees working in these different hospitality workplaces ranged from 12 to 40. Hospitality workplaces, such as hotels, bars, restaurants, fast-food establishments, cafeterias, and taverns, were chosen as the setting for this research. Hospitality jobs are customer service positions in hotels, restaurants, events, and other tourism industry areas. The hospitality workplaces where the participants were recruited were hotels, bars, restaurants, fast-food establishments, and cafeterias.

### Study design

An exploratory qualitative design was also conducted. In-depth interviews (IDIs) and focus group discussions (FGDs) were conducted to explore the individual and group perspectives of WSH’s experiences during work. On the other hand, key informant interviews (KIIs) were conducted to gain an in-depth understanding of the WSH’s issue at work from hospitality workplace supervisors/managers, cashiers, and customers.

### Study participants

Women employees who had at least 6-months of working experience in the hospitality industry and experienced workplace sexual harassment while serving in the workplace within the last 6 months were included in this study. The participants worked in hospitality workplaces in the study area. After identifying the first women, women who worked in hospitality workplaces and experienced WSH were identified and contacted using the snowball method. Non-governmental organisations’ community workers living in the city where the study participants live help us reach them. Further, Key informants were recruited purposefully to gather evidence that supplements women employees’ ideas. The enrolment of the study participants was continued until the data was saturated. Those customer key-informants who had a physical and mental illness that deterred their communication ability were excluded from the study.

### Sample size and sampling techniques

Ten IDIs and six FGDs were conducted with female employees to understand their experiences. A total of 35 female employees participated in the focus group discussions. Two FGDs each had five participants, one FGD had seven, and three FGDs each had six. The additional sampling progression was stopped based on information saturation. Based on a criteria-based purposeful sampling, 13 KIIs (five male managers, four female cashiers, and four male customers) were selected and interviewed. The selection criteria were serving more than 6 months in a hospitality workplace with more than six female employees (managers, cashiers) and those perceived as regular customers by the employees.

### Data collection

Data were collected from 1 January to  30 August 2019. The information was gathered through a variety of methods and sources. For a more in-depth understanding of the phenomenon, several data collection approaches have been used.

The interview guides were intended to have discussions with the study participants. The participants’ questions to elicit the WSH experience were: whether they had faced any activities that made them uncomfortable. The guides for IDIs and FGDs were similar, but a distinct interview guide was developed for KIIs. The issues discussed in IDIs and FGDs were women’s views and perspectives, while the issues discussed in KIIs were for a more in-depth understanding of the phenomenon. Issues, such as potential risk factors and effects, were posed and included during extensive conversations with employees and key informants (managers, co-workers, and customers).

Furthermore, the perceived impact of WSH was included. For the interview questions’ consistency and correctness, all guides were first prepared in English, translated into Amharic (the local language), then back-translated and rechecked by a third person. All guides were pre-tested on five women employed in hospitality workplaces with similar demographic profiles. The pre-test was planned to ensure their suitability for improving the guidelines and interview techniques for the local setting. These participants were not included in this study.

All discussions were conducted in Amharic, a local language. In-depth Interviews and FGDs with women were conducted in a convenient place for the study participants. The IDIs and FGDs were held in a hotel where female workers felt comfortable and secure. The study participants wanted to ward off their work surroundings to have free discussions about their perception, work experience, and impact. The researchers also wanted to evade the formality of the hospitality workplace environment. The researchers conducted the FGDs in a way that was hired to perform the treatment safely and competently. The researchers also tried to make the location an average place where all participants could access transport.

Key informant interviews were conducted in a private room voluntarily provided by the hospitality workplace Managers and supervisors. Appointments were made over the phone for each participant. Four researchers (first author (male) and three female qualitative experts) conducted the FGDs and IDIs (two for each): one facilitated the discussions. At the same time, the other assisted with getting together the women and taking notes as required. With the participants’ permission, aAll interviews were audio-recorded with the participant's permission . The duration of each interview and focus group discussion ranged from 60 to 105 min. The participants were provided with tea, coffee, water, soft drinks, and transportation costs. FGDs and IDIs took place at all hours of the day and at night (until 8:00 PM).

### Data analysis

All recorded interviews, FGDs, and field notes were transcribed verbatim to Amharic (the local language) and then translated into English. The transcripts were prepared by a research assistant who was a university graduate with experience in conducting qualitative research and the first author. Twelve (40%) (three FGDs, five IDIs, and four KIIs) of the transcripts were cross-checked with audio files to ensure accuracy and consistency before coding. The first Author (MD) reads a sub-sample of transcripts (prepared by the expert) to check for consistency. Data were analysed using Braun and Clark’s (2006) thematic analysis approach [64, 65]. To take the thematic analysis, the team re-read the descriptive information to become intimate with the facts to obtain codes for thematic analysis. The analysis approach is based on data-driven codes. Data-driven codes were performed using an open coding method, which included categorising small codes. To ensure the reliability of the coding, the principal investigator and co-investigators independently coded a set of transcripts from each interview category reached a consensus on a list of codes and had all authors verified it. When there were disputes about  the nature of the codes, the study team had talked about finding a consensus on the final code list and an interpretation agreement. The codes were added to the subsequent transcripts using the computer software Atlas-ti, version 8.4. Next, the first author grouped the small codes to generate main themes, which were then debated, decided upon, and checked by the team, with emerging themes becoming the categories for analysis [66].. These key themes provide a basis for the thematic framework. These ideas were produced through analytic thinking as new ideas were identified inductively from the data.

### Data quality management and assurance

In addition to the techniques performed under each activity, different techniques were considered to ensure the study’s credibility, dependability, transferability, and conformability. After a pre-test was conducted among participants with a similar population and setting, the interview and FGD guides were edited and modified by qualitative research experts. Second, the facilitators of the FGDs and IDIs, and two key informants (supervisors) were invited to check the correct representation of the study’s findings and ideas. Third, to increase the credibility of the findings, the team triangulated the data collected from female hospitality employees, supervisors, cashiers, and customers. Then, to check the consistency between the analysed data and the last textual findings, the research team invited the people participating in the interview and focus group discussion, sent the transcriptions by email, and received comments from them. Moreover, respondent bias and the risk of reactivity were ensured by holding back researchers’ predetermined ideas about the issue under study.

## Results

### Sociodemographic characteristics

Fifty-eight participants (45 female employees, five managers, four cashiers, and four customers) participated in the six FGDs, ten IDIs, and thirteen KIIs. The average length of record for each IDI and FGD was 80 min and 40 min for KIIs. The female'sage was from 18 to 37 years. The key informants involved managers, cashiers, and customers who work as merchants, tour guides, and drivers (Tables 1 and 2).

**Table 1:** Sociodemographic profile of women hospitality workplace employees involved in the focus group discussion and in-depth interviews in Bahir Dar, January to August 2019 (*n* = 45)

	IDIs (*n* = 10)	FGDs (*n* = 35)
**Mean age (SD)**	24.4(±4.88)	24.83 (±3.30)
**Educational status**		
Primary education	1	4
Secondary education	8	28
College and above	1	3
**Mean years (SD) of experience**	2.8(2.15)	2.42 (±1.64)
**Raised area**		
Urban	3	26
Rural	7	9

*IDIs* in-depth interviews, *FGDs* focus group discussions, *SD* Standard deviation

**Table 2:** Background information of the Key informants involving in in-depth interviews with supervisors, cashiers, and customers of hospitality workplaces in Bahir Dar, January to August 2019 (*n* = 13)

ID	Profession/position	Educational level	Experience (year/s)
KI1	Cashier	10th grade	2
KI2	Cashier	10th grade	5
KI3	Cashier	8th grade	6
KI4	Cashier	Degree holder	1
KI5	Merchant	4th grade	NA
KI6	Driver	10th grade	NA
KI7	Driver	10th grade	NA
KI8	Tour guider	10th grade	NA
KI9	Supervisor	Diploma holder	2
KI10	Manager	Degree holder	6
KI11	Manager	Degree holder	7
KI12	Manager	Degree holder	10
KI13	Manager	Degree holder	4

*NA* not applicable, *KI* Key informant

Four themes and fifteen sub-themes were covered identified in this article. The identified themes include (1) the perception of WSH, (2) the experience of WSH, (3) perceived risk factors for WSH victimisation, and (4) consequences of WSH victimisation.

### Perception of sexual harassment

All the participants perceived that sexual harassment as a common issue in their workplaces. The subthemes under this theme were pressuring, threatening, touching, and abducting for sexual advances. Even though they did not classify them into distinct categories, they perceived different sexual harassment incidents in hospitality workplaces.

#### Pressuring for sexual advances

Most of the participants perceived that sexual harassment is being pressured to engage in unwanted sexual activities through tricks, including exaggerated tips and inappropriate promises of rewards in exchange for sexual favours:

*“Sexual harassment is a condition in which women working in [hospitality workplaces] are pressured to do sexual activities without their will. Mostly, they may be tricked through tips, another unnecessary gift, or inappropriate promise of rewards in exchange for sexual favours.” (25 years, IDI, four years of experience in a cafeteria).*

Other incidents that the participants perceived as sexual harassment were activities conducted by the supervisors or the owner. These activities include promoting and offering a new job and giving money in exchange for sexual favours:

*“Sexual harassment is the supervisors’ or owners’ action that can be explained by providing money, and promising rewards, and promoting for a better job situation with a better salary scale in exchange for advanced sexual favours.” (FGD, two years of experience in a restaurant).*

#### Threatening for a sexual advance

The participants also perceived that sexual harassment was the activity of the sexual perpetrator that was expressed by threatening to hurt women’s relatives, firing from a job, complaining or falsely accusing about the provided service to the immediate supervisors in exchange for sexual favours:

*“Sexual harassment is identifying the women’s weak side that makes it difficult to overcome the sexual requests. The soft parts could be her financial problem, her relative, or her beloved one. So, I think sexual harassment is expressed by threatening to hurt her relative or beloved one, complaining about her service provision performance to her immediate boss, threatening to fire her from a job, and not paying for the services unless we accept his sex requests.*” *(IDI, four years of experience in a cafeteria).*

#### Touching sensitive parts of the body

Furthermore, participants perceived that sexual harassment was expressed by touching sexually sensitive parts of women, random sexual jokes, verbal sexual requests, repeated requests to sexual mating, sexual solicitation, sexual intimidation, sexual prodding, and requesting telephone number:

*“Touching the breasts, hips, and genitalia, slapping the hips and the face, requesting sexual intercourse, commenting on physical attributes, and inviting dining and requesting sex are some of the things at which sexual harassment can be explained.” (IDI, 1-year experience in a cafeteria).*

Similarly, participants perceived that showing pornographic movies/pictures, writing sexual messages on the pay bill, unfair treatment of women, and undermining the women were the parts of sexual harassment:

*“I believe sexual harassment is explained by … , winking, and undermining me considering my gender.” (FGD, two years experience in a restaurant).*

#### Abducting for sexual intercourse

Lastly, participants perceived that sexual harassments was expressed through abducting, raping, slapping, kicking, pinching, and verbal insult of the women:

*“Oh! I think sexual harassment is rape or abduction.” (FGD, four years of experience in a cafeteria).*

Another participant added:

“*… sexual harassment could be explained by spitting of drinks, slapping, pinching, caressing, talking unnecessary sexual talks, and talking and distributing false things about me to the manager.” (FGD, five years of experience in a cafeteria).*

### Experiences of sexual harassment

Besides their perception, women recognised a variety of incidents in their workplaces. The subthemes under this theme include verbal, non-verbal, and physical types of workplace sexual harassment and perpetrators. The participants noted that the perpetrators were agents, colleagues, customers, supervisors, and owners. Although they did not categorise the incidents, the research team classified their experiences as verbal, non-verbal, and physical types of sexual harassment.

#### Verbal experience of sexual harassment

The verbal forms of sexual harassment experiences include cat-calling – whistling, yelling sexually suggestive comments, usually at a stranger; unwanted flirting; and jokes referring to sexual acts and sexual orientation. It also includes unwelcome graphic comments about a person’s body; unwelcome and inappropriate inquiries about a person’s sex life; sexual favours – asking for sexual favours from a co-worker or peer; and other sexual advancements. Participants in this study reported that women were often harassed often in hotels, restaurants, cafeterias, and groceries and feel uncomfortable. They also reported that female workers experienced the threats of firing from a job, hating relatives/beloved, accused her of improper service provision in exchange for sexual favours. Their female co-workers were reported to accept perpetrators sexual requests out of fear of retaliation if they turned away their unwanted sexual overtures:

*“When we refuse to give our phone number to them, they will call the manager and falsely accuses us of not serving them properly. If we explain ourselves as we had a husband, children, and family, they will put the bill bag upside down.” (FGD, two years of experience in a cafeteria).*

Other participants added:

*“When I was in a bar, unwanted sexual acts such as fondling, undermining, pushing us towards undesirable sexual acts using money and intimidating. The perpetrators did not realise that we were working for survival. As per their understanding, we all are doing transactional sex to get money from them.” (IDI, 1-year of experience in a bars).*

*“.... I experienced many things regarding sexual harassment. Some customers spit on me, fondles me, kicked my hip, touched my breasts, and tried to kiss me forcefully. Some also wait for me after I finished my job and threatened me to spend the night with them and engaged in sexual intercourse with them. Generally, it is the workplace where we gain when we are unable to get another option.” (IDI, four years  ofexperience in a restaurant).*

Participants reported that they experienced inappropriate promises and too many tips to accept sexual requests. They also mentioned that they experienced comments about their physical attributes, requests for dates, requests for telephone numbers, requests for sexual advances, and verbal insults while they are at their job:

*“Ha … ha … ha … [starts to laugh] … Then, he asked me to eat dinner with him, requested me to spend the night with him, and asked me to have sexual intercourse with him.” (IDI, three years of experience in a lodge cafeteria).*

Another participant added:

*“On one occasion, a famous and rich man approached me. He has been my customer, and he mostly gave  me an exaggerated tip for me. He has a marriage ring on his finger. Mostly he had been with his friends. Only later, sometime, he started to become lonely. I served him as usual. … One day, he requested my telephone number, called for me, and requested me to accompany my dwelling house. I did not hesitate; I handed him my telephone number. He called me later at night. I talked to him in detail. He informed me that he was not happy with his spousal relationship. He told me that he could change my life. He also promised to open a business centre and invited me to have sex with him. Merely, I turned down upon his request. Also, I told him to keep confidential what he requested.” (IDI, two years of service in a cafeteria).*

#### Nonverbal experience of sexual harassment

The non-verbal forms of sexual harassment experiences were unwelcome gestures of a sexual nature – looking someone up and down in a way that makes that person feel uncomfortable, blocking someone’s path; indecent exposure (e.g., “flashing”); and unwelcome display and sharing of sexually explicit pictures and objects. This form of sexual harassment was prominent in the participants’ discussions. Accounts of nonverbal sexual harassment experiences were overt or covert sexual pressure, such as winking, showing pornographic movies, pictures, undermining the women, unfair treatment, gazing, and composing messages on the bill:

*“… Leaving their phone number on the bill, winking, gazing, and so on.” (FGD, two years of experience in a cafeteria).*

Another participant added:

*“Most of the things that I experienced in the hospitality workplaces are … , showing pornography movies, writing a message on the bill, winking, … , and other gestural signals.”(IDI, two years of experience as a waitress in a hotel).*

#### Physical experiences of sexual harassment

The physical forms of sexual harassment experiences include unwanted touching or physical contact (e.g., an arm around the shoulder; a hand placed on a thigh or another part of the body; standing up against someone after being told to move away); and being subjected to a strip search of the opposite sex presence. Participants reported the types of sexual harassment experiences: abduction, fighting to kiss, fondling, forcing to do sex without willingness, kicking, pinching, slapping, rape, and touching the erogenous part of the bodysensitive areas.

*“Most of the activities I experienced in the hospitality workplaces are, … , pinching, fondling, touching the buttocks and the breast. There are also winking, … , and other gestural signals of erotic request.” (IDI, two years of service in bars).*

Another participant added:

*“…*. *Some came to the organisation for the first time, touched my breast, touched and slapped my hips, fondle me, forced me to kiss, and touched my sensitive sexual parts, …*.” *(21 years, IDI, two years of experience in a cafeteria).*

#### Who is responsible for the perpetration?

Participants sought the responsible bodies of sexual harassment perpetration. These categories include customers, supervisors/managers/owners, male co-workers, agents (brokers), and transactional sex workers. Under the customers’ category, they mentioned different people with diverse professions. However, they emphasise that the incident was worse among wealthy elderly adult customers:

*“The local old and wealthy guys are a more challenging group. …. They tried to take us to very unusual places … , which is far from the populated area.” (FGD, three years of experience in a cafeteria).*

Other participants added:

*“Mostly married and older adults are the perpetrators. Those people, for the most part, went to restaurants to recruit girls for sexual harassment. They ordered something and did not use what they ordered. Instead, they harassed us and asked for sexual intercourse. We know them, but they removed their marriage ring and coming to us, simulating that they did not marry.” (FGD, three years experience in a cafeteria).*

*“Old men like me just shaved their beard, have brokers whom they will bring girls from hospitality workplaces and universities. Brokers are doing senseless work. The low-income family sends their daughters to work and universities, but brokers bribing women and girls and sell them to elderly people whom they want to have sex with.” (Customer, KII, Merchant).*

The managers/supervisors/owner’s category was the second of the mentioned categories:

*“When we apply for the job as waitresses, the first question which is going to be asked by the manager or supervisor is not educational status, and it is not the work experience; it is a willingness to have sex with him.” (FGD, six years of experience in a restaurant).*

Another participant added:

*“The head waitresses also harass us, and receptions/cashiers are the sources of our address for the perpetrators. The receptions and cashiers will be given our address to get money and other incentives.” (FGD, three years experience in a bar).*

Conversely, transactional sex workers’ presence and their way of attracting customers also lead to the perception that all employees are engaged in such activities. Besides, per their description, some women were working as an agent to create a relationship:

*There are two types of waitresses. The first group is women who have family, marriage, and children. This group needs their job and help their family. The second group is young women who have no family. This group mostly does transactional sex for covering their expenses to rent a home, buy food, cosmetics & cloth, and sometimes create a link between the perpetrators and the victims. By the way, this is because of the lower salary paid by the hospitality workplaces. Perpetrators considered all waitresses like the second group.” (IDI, two years of experience in a cafeteria).*

### Perceived risk factors of sexual harassment

Women, customers, cashiers, and managers noted various factors that place women working in the hospitality workplace at risk for sexual harassment, including factors related to customers, victims (women), organisation, and others (Society, peer, and policy-related risk factors).

#### Customer-related factors

Participants mentioned that the customers’ perceptions, such as considering women as a transactional sex worker, commercial sex worker, ordinary object, interested in related sexual matters, and easy-to-get employees for sex:

*“The customers perceive that all waitresses are transactional sex workers or commercial sex workers, and they ask us to have sex with them using their money.” (IDI, two years experience in a restaurant).*

Another person added:

*“One of the driving factors is that most of the waitresses engaged in commercial sex work and customers perceive that all are interested in this work.” (FGD, five years experience as a waitress).*

It was mentioned that customers’ behaviours, such as being alcoholic, being sex addicted, and failing to set up successful spousal relationships, were also among the risk factors for sexual harassment:

*“Sometimes, I think that they are addicted to having sex. Since they are married, they can get sex with their wives. However, they came to us to do the same thing with their money. Most of the married perpetrators try to convince me that cheating is healthy and has no problem.” (IDI, two years experience as a waitress).*

Similarly, some respondents also mentioned that the activities of the customers such as threatening to harm relatives/beloved, undermining the work or the workers, and provision of an exaggerated tip in exchange for sexual favours:

*“Perpetrators approach the women and identify the women’s weak side to get an easy way for their request. The weak parts of most women are finance/money, relatives, or their darlings. As a result, the perpetrators threaten us to agree to their sexual requests, or they will harm our relatives/darlings and will not pay for the services that they used.” (FGD, three years experience in a cafeteria).*

Another participant added:

*“After all, in a big hotel or small catering, waitressing is considered less critical work by the customers. Some waitresses are also considered non-civilised. Rarely do some customers only understand the job and the workers. However, some classified us as commercial sex workers.” (FGD, three years experience in a bar).*

#### Victim-related factors

Participants reported that women deliberately pursue a relationship established upon the male providing financial help. Then some men expect sexual advances in return for financial help. This act implies that women rely on income from customers, poverty and financial problems were the risk factors for WSH:

*“… Due to the lower salary, we sometimes engaged in sexual activity for the compensation of our economic problems through the perpetrator’s money.” (FGD, two years experience as a waitress).*

Participants perceived that women who came from a rural area, young and inexperienced waitresses, and women with low awareness of the hospitality environment/sexual harassment are most vulnerable. It was reported that the pre-work awareness created in each organisation were instructions about customer handling, wearing styles, work, salary, and Organogram of the workplaces. Some women reported that they had got training related to sexually transmitted infections, including HIV. However, they mentioned that they were not aware of WSH:

*“Most of the time, they gave direction about customer handling and other issues that affect their business. Sexual harassment is not their business.” (FGD, two years experience in a bar).*

Another participant added:

*“The pre-work instruction document in hospitality workplaces states all about time management, uniform, customer handling, and others that mainly to maximise their benefit. It is not mentioning anything to keep the right of the women working in these workplaces.” (FGD, four years experience in a restaurant).*

Customer handling style, frequent contact with customers, the beauty of the women, and gender norms are the other perceived risks of sexual harassment by the participants:

*“We are expected to be very friendly and communicative for customers. However, this may lead to a casual relationship.” (FGD, three years experience in a bar).*

Another participant added:

*“… , women’s natural beauty and their welcoming approach makes them more vulnerable to sexual harassment.” (KII (customer), driver).*

Behaviours of women working in the hospitality workplaces were also viewed as increasing their WSH’s risk, including transactional sex for financial support. Women and the key informants reported that some women set up relations with men to support themselves financially:

*..., young women who do not have a family have transactional sexual relations to cover their expenses related to home, rent, food, cosmetics and clothing. By the way, this is because of the lower salary paid by the hospitality workplaces. Perpetrators considered all waitresses like the second group and did sexual harassment to all waitresses.” (IDI, two years experience in the cafeteria).*

Some women also create relationships with customers for a specific purpose. They tried to accept invitations, call customers with a nickname, chew gum in front of the customers, show different walking styles and gratuity (Amharic-gursha). They also keep silent while a customer touches their sensitive body parts, touch the customers back, laugh unnecessarily:

*“Chewing gum, accepting dinner, and other invitations by the customer indicates desire.” (FGD, four years experience in a restaurant).*

Another participant added:

*“The signs of the willingness of the waitresses, such as, willing to gratuitous, unnecessary laughing, feeling the customers back or face, and nicking is also the driving factors.” (IDI, two years experience in a restaurant).*

#### Organisation-related factors

Participants respond that some organisations encourage women to wear uniforms or clothing that show their body (breasts, buttocks), encouraged to wear sexually attractive clothing, and encouraged to wear uniforms that show their upper legs:

*“Some of the [hospitality organisations] need to make the waitresses a sexual object. They dress them in short /miniskirts/ that exposed their body. The uniform is attractive, which can display their collection for attracting customers sexually.” (IDI, four years experience in a cafeteria).*

Similarly, respondents noted that the nature of the job, night shifting, perceiving that a customer is a king and always right” were the factors that expose women to different forms of sexual harassment:

*“Practically, we women are victims. Whereas customers are considered as kings and always right. I faced such a problem while I complain of sexual harassment to my manager.” (FGD, five years experience in a cafeteria).*

On the other hand, lack of a fixed salary scale or small salary, lack of grievance management, and rules and regulations in the hospitality workplaces were also the risk factors:

*“The monthly salary for women working in hospitality workplaces is not enough. They cannot afford a dorm, food … , and it has a Burdon on them. To overcome this time, women will negotiate with the perpetrators to get money in exchange for sexual favours.” (KII (cashier), 1-year experience in a restaurant).*

Another participant added:

*“So far, I did not know organisations working to address such problems and have a formal complaints procedure. There are no special rules and regulation for privileged waitresses safety of sexual harassment in their working place.” (FGD, five years experience in a restaurant).*

Manager’s power and influence were also the other organisation-related risk factors in some hospitality workplaces:

*“Managers/supervisors/owners tried to use their power to harass sexually. If we are not volunteers, they will fire us from the job. If we are volunteers, they will promote to head waiter from an ordinary waitress in exchange for sexual favours.” (FGD, four years experience in a cafeteria).*

#### Society, peer, and policy-related risk factors

Society’s perception, peer sexual pressure, and lack of governmental/professional association which could work on hospitality employee’s sexual and reproductive issues:

*“For those who need to complain, the statutory institutions want witnesses, and it is unlikely to get any solution for acts such as touching, winking, and fondling. Those who see this act are unwilling to witness it due to the fear of not being fired. Even with the presence of a witness, we are not ready to file a complaint. It is due to the long process of the complaint. Mostly, we thought that the legal process takes time and money. The legal bodies act if they see someone is hitting us. I do not think that there is a legal issue with sexual harassment. I think the legal process is not giving solutions.” (IDI, 1-year experience in a cafeteria).*

Further, the participants also noted drivers, such as agents (brokers), culture, corruption, easy accessibility of women who could work in hospitality workplaces:

*“If someone raped me, I would do nothing. Sometimes, agents/brokers, either the perpetrators or the dealers of the activities. So, it is so difficult to solve the issue with the legal ground. Most of the perpetrators can be able to stop the case with money. Therefore, since it is not simple to take the issue to court, I will not go to court because no one will consider the issue.” (FGD, three years experience in a restaurant).*

Another participant added:

*“It is known that waitresses are targeted for sexual harassment because of our culture, physical beauty, easy-obtainability, and financial problems.” (KII, manager).*

### Consequences of sexual harassment

All participants tried to delineate categories of consequences of sexual harassment. These were work-related, health-related (mental health, reproductive health, and reproductive health), family undermining, and financial consequences.

#### Work-related consequences:

Participants reported that job-hop, including changing the locality, work withdrawal (lateness, absentees), and being a coffee seller nearby the roads are the effects of frequent sexual harassment in the hospitality workplaces:

*“It depends. Some may deteriorate. However, some may take victimisation as a sprinting for future life. Some may end in … , and in coffee selling in the street. However, some change their jobs or marry a rich person and become stable in their marriage.” (IDI, 1-year experience in the cafeteria).*

Another participant added:

*“Once I prefer not to suffer from frequent sexual harassment. I searched and got another waitressing job in another institution. However, it was the same. Then, I changed my job to the barber.” (IDI, one and half-year experience in a bar).*

Similarly, participants noted that the organisations were failing to have productive workers and lose their dignity due to the frequent sexual harassment occurring in them:

*“… It can reduce the organisation’s image, dignity, and community acceptance. It also leads to the loss of productive working group women.” (IDI, 1-year experience in a cafeteria).*

Moreover, women employees in the hospitality workplaces reported that they sometimes faced job stress, job dissatisfaction, work disrespect, lack of promotion for a better situation, and hated the job due to the frequent sexual harassment in some hospitality workplaces.

Some participants reported that they were promoted to a better position, got good recommendation letters, and got married as a result of the harassment they faced and the response they gave (agreement) to the perpetrators:

*“The consequences are different. However, some may take the victimisation as a sprinting for their future life and maybe promoted to a better job position, get better work recommendations in exchange for sexual favours, marry a rich person, and have a stable life.” (IDI, four years experience in a cafeteria).*

#### Health-related consequences

##### Mental and behavioural consequences:

Almost all the participants reported that women working in hospitality workplaces faced mental and behavioural, physical, and reproductive health consequences due to frequent sexual harassment. Participants remarked that the hospitality workplaces’ frequent sexual harassment affected their well-being (psychological, physical, and social (relationship)).

They noted that they felt depressed, not enjoying life, not optimistic about their future, and failed to control their lives. Again, they noted that they were distressed with their life, felt sad, failed to survive the way they want, lack self-confidence, lack self-esteem, and felt hopeless about their future:

*“…. I considered myself a person who has no value, lost my confidence, thought about suicide, and felt sick of the frequent acts. I hate my work myself and felt that working in hospitality workplaces is a disgrace. I asked myself, how does the perpetrator sexually harass me without recognising me? Sometimes I just cried. I also thought about my boyfriend’s thoughts.” (IDI, 1-year experience in a bar).*

They also reported that they felt unhealthy physically, saw terrible dreams at night, dissatisfied with their daily activities and lacks adequate money to live, hate what they are trifling, and lack the cliches to work in hospitality workplaces:

*“I realised that the waitresses lose their trust. They lose their interest in working and discuss their issues. They develop fear and lack of self-confidence, moral disengagement, psychological depression. Then, they withdraw the job, depression, lack of self-esteem, fewer clutches to work.” (KII (supervisor), two years experience).*

Furthermore, they reported that they felt helpless, socially isolated and ignorant, blame themselves, hate themselves, addicted to substances/smoke, careless, felt useless and powerless, lack trust, question themselves, and felt ashamed/shy:

*“The perpetrators affected my life so badly. I felt guilty, shameful, depressed, and fear every human beings while I moved home with transport, and suffered a terrible dream at night.” (IDI, four years experience in a cafeteria).*

Conversely, some reported that they were engaged and built a successful career as a result of the relationship that started with sexual harassment:

*“However, some change their jobs or marry a rich person and become stable in their marriage. As a consequence, they could receive a good future.” (IDI, three years experience in a restaurant).*

Lastly, some reported that they felt depression, anxiety, stress, had suicidal ideation, and psychological trauma as a result of sexual harassment that happened to them in the hospitality workplaces:

*“All these activities make me hate the job and expose me to depression, fear, and self-hate. I feel angry, disturbed, think that I am not a person like others.” (IDI, two years experience in a bar).*

##### Physical health consequences:

Some of the participants reported that they were bruised, injured, developed headache, fatigue, and other physical complication (fistula) as a result of some physical forms of sexual harassment:

*“… Seven individuals have captured me at a time. Nevertheless, the police saved me though they beat me. I shed blood while he kicked me with his ring worn hand. I lost my phone, necklace, and tip. I also knew a female who faces similar situations. Eight adult individuals had violated her, and she got faint while the 9th individual had started climbing her. After then, she took the illness. Presently, in that respect, is a leakage of fluid from her genital area.” (IDI, six years experience in a cafeteria).*

##### Reproductive health consequences:

Participants also mentioned the connection between their WSH experience and their reproductive health problems. They reported developing menstrual disorders, participating in sex trade/transactions, abortions, unintended pregnancies and acquired sexually transmitted infections (STIs), including HIV/AIDS:

*“Since we may not receive what we wish to have, in this instance, we may practice transactional sex and other social issues. I knew one young woman who was a waitress first and then became a commercial sex worker. This involvement in commercial sex work is one problem.” (FGD, six years experience in a cafeteria).*

Other participant added:

*“Sexual harassment is one of the reasons for exposure to HIV/AIDS … , and would be exposed to stigma and discrimination.” (FGD, two years experience in a restaurant).*

*“Mostly physical and psychological impacts such as depression, menstrual disorder, tiredness, and fear happened to me.” (IDI, two years experience in a bar).*

##### Financial and family undermining consequences:

Participants reported that they faced financial problems and family undermining after they were victimised by sexual harassment in the hospitality workplaces:

*“The anger that happened in my workplace due to the unwanted sexual acts made my display on my family and disturbed my family relationship.” (IDI, two years experience in a restaurant).*

*“Frequent sexual harassment leads to job-hop, unwanted pregnancy, and to encounter a different financial crisis, social stigma, HIV/AIDS, and street life.” (KII (customer), driver).*

## Discussion

This research used the IDIs, FGDs, and KIIs to discover that WSH was common among women working in the hospitality industry. It also showed that women’s perceptions of WSH were distorted. All of the study’s participants agreed that women employed in the hospitality industry face various types of WSH, including verbal, nonverbal, and physical harassment. This study also established several factors that must be tackled to bring about practical change in Ethiopian hospitality workplaces and the workplace sexual harassment-related implications, consistent with other studies conducted in the hospitality industry [10, 67]. These findings show that despite the prohibition through a criminal code proclamation, prescription of simple imprisonment for the perpetrator [53], and considered a prohibited workplace act [54], WSH is a concern in Ethiopia.

Furthermore, some of the participants in our study had a clear understanding of the idea of sexual harassment, which is consistent with earlier studies [68]. However, most respondents had inadequate awareness or training about WSH before or during their employment process, confused about differentiating WSH, ambient harassment, and violence. This finding was consistent with the Zimbabwean study [8]. It also indicates that women employees in hospitality workplaces were not recruited based on merit. This finding indicates that though women workers were required to have awareness and skills in managing WSH beyond the hospitality workplaces, the lack of exposure to different sexual and reproductive health-related training, including WSH, make them fail to differentiate WSH from the other forms of violence. Thus, to be aware of WSH in hospitality workplaces, a set of unique approaches and system reforms must be introduced to enhance women’s employees’ knowledge and working capacity. These approaches must introduce training for women employees to increase confidence in preventing WSH at their workplaces. The training should be in pre-service education regularly, in-service training, and professional development. There should be curricula, pre-service training and accredited by the Ministry of Science and Higher Education of Ethiopia. In-service and professional development training should also include induction or orientation training, foundation training, job training, refresher or maintenance training, and career training. Further, awareness should be created for both the victims and perpetrators using different behavioural change communication and information, education, and communication approaches. In this approach, posters that can create awareness of the service users could be helpful.

Furthermore, in this study, participants responded that they had experienced verbal, physical, and non-verbal forms of WSH, which align with the findings in South Africa’s [69] and Australia [70]. The verbal forms include comments about physical attributes, lustful calls, threats in exchange for sexual favours, tips & promises in exchange for sexual favours, dirty sexual jokes/stories, frequent requests for dates, verbal insult by the perpetrators targeting their sexual orientation, targeted for rumours of sexual promiscuity, and offering money in exchange for sexual favours. Additionally, WSH’s physical forms were touching, unwanted kissing, violent sexual acts (rape, holding hands and clothes, hugging), staring at breasts and hips, fondling, and cornered or placed in a position was difficult to get out. Workplace sexual harassment’s non-verbal forms also included seeing perpetrators watching pornographic pictures, receiving a love letter, and gesture requests of sex (i.e., winking, gazing, leering, ogling, and staring). Furthermore, gender-related demands were discouraging because of being a female, unfair treatment, forcing to give sexual services like sitting beside the perpetrators and wearing uniforms that provoke sexual desire. The perpetrators also initiated the women to sexual advances in exchange for job employment, recommendations, and a better job position. As a result, in line with a study conducted among Mexican indigenous farmworker women in Oregon [71], women in this study reported that these experiences made the hospitality workplaces feel unsafe and unfair. Thus, hospitality workplace management should try to control specific unsafe acts by eliminating unsafe working conditions and implementing government proclamations. Organisations also should prioritise risk factors and pay more attention to control them to achieve a safer working environment.

The perpetrators of WSH mentioned above were customers, co-workers, and immediate bosses (supervisors/managers/owners), which is consistent with the findings of studies conducted in Zimbabwe [8] and the USA [10]. On the contrary, unlike other studies, the study participants emphasised that some women and agents (brokers) were also responsible for the act. This finding supports the statement that emphasises peers’ more considerable influence than managing labour sexualisation [72]. This covert and overt involvement of hospitality workplace managers and co-workers/peers, and agents (brokers) in the perpetration act made the problem further complicated. Thus, the stakeholders such as the Ministry of labour and social affairs of Ethiopia, the Ministry of culture and tourism, and the Ministry of health, together with non-governmental organisations, should give attention, set ethical standards in hospitality workplaces, and provide ethical guidelines for employees that focus on WSH. These ethical standards and guidelines should influence employees’ ethical behaviours and identify appropriate ethical judgment in the workplace. These stakeholders also should establish strategies to monitor the implementation of those ethical standards and guidelines. However, the indication of women’s hidden perpetrators in the employment process (i.e., the agents, who introduce individual employees to an employer, also request sexual advances to introduce them to the employer) was an essential and unique finding that needs further empirical studies on the issue in different contexts and occupations. Organisations should, however, consider this group of individuals while they are giving orientation to their employees.

Also, some women in the current study practised transactional sex. This finding is consistent with a study conducted in Cameroon [73]. In line with other studies [50, 51], transactional sex practice was due to the low wages inadequate to fulfil basic needs and improve social status. These women showed attention-seeking behaviours and displayed an interest in creating a relationship with service users. These attention-seeking and relationship creation practices include accepting invitations, calling customers with a nickname, chewing gum in front of the customers, different walking styles, touching customers, and taking a perk. These practices created a perceptual experience that all the women working in hospitality workplaces have the desire. Thus, in line with a study conducted among university students in Ethiopia [74], transactional sex practice is considered a risk factor for WSH in hospitality workplaces. These practices, their engagements in commercial sex work, and STIs/HIV were also the reported reproductive health effects of WSH. These findings align with other studies [75–80] and could be due to this practice’s risky nature. These findings imply that some women’s transactional sex practice was either a risk factor or WSH’s effect in hospitality workplaces. It also indicates that there were indirect sex workers who did not get attention from health authorities and could be reasons for high STIs reports, including HIV/AIDS among frontline service workers of the hospitality workplaces. Hence, there is a necessity to study the magnitude and plan schemes to reject or dilute the problem.

Participants in this study also perceive WSH victimisation factors such as the organisations, the customers, the women working in the hospitality workplaces, society, legal bodies, agents, culture, and corruption. Consistent with a study conducted among restaurant workers in Canada [81], employer hiring practices and clothing codes that emphasise physical attractiveness, the customer-service orientation of hospitality workplaces, and customers’ involvement with tips create an environment that exposes women to WSH. Like other hospitality industry sexual harassment studies [31, 32, 82], organisations in this study hired attractive and young women, failed to orient their employees about WSH, created sexually objectifying environments, tolerated WSH, accepted that WSH is inevitable, and perceived customers as kings. Additionally, this study found that employees’ practice of a transactional sexual relationship or acting as a commercial sex worker, perpetrators’ behaviours, such as sex addiction, alcohol addiction, and unhappy marital relationships of customers, were the perceived exposing factors for WSH. Likewise, perpetrators’ threats (to hurt the women’s relatives, fired from a job, and demoted from a better position) and provision of tips, promises, rewards, promotion, and work recommendations in exchange for sexual favours were also perceived exposing factors. In line with other studies [83–85], these findings imply multiple risk factors for sexual harassment in hospitality workplaces. Thus, employers must note that WSH is a warning sign threatening workplace productivity and a stable workforce. Future research should also consider a multi-level study incorporating organisational perspectives such as power distance, workplace culture, job-gender context, and individual perspectives such as personality traits, personal characteristics, and socio-economic status.

Furthermore, this study participant experienced work-related, health-related (i.e., mental and behavioural health, physical health, and reproductive health), economic, and family-related consequences. This result agrees with findings from other workplace studies [48, 49, 63, 86–91]. Like the other studies, the possible reasons might be the peculiarities of the industry, such as customer power [13, 72, 92], the sexualisation of the workplaces [31, 32], workplace culture [93], and sociodemographic characteristics [94] of women as risk factors of WSH. Consequently, the peculiarities mentioned above might end in different work-related consequences [12, 95]. Thus, in line with the findings mentioned above, in this study, the frequently stated work-related consequences include work withdrawal, job withdrawal, lack of motivation, job stress, and job dissatisfaction. Furthermore, consistent with other studies [48, 66, 69], participants in this study reported that WSH affects their general well-being. The results of this study’s depressive symptoms, anxiety, tension, and post-traumatic stress symptoms were similar to those of studies conducted in Ethiopia among female university students [61] and female faculty and staff [62] in Ethiopia. Participants in this study have documented physical injury, headache, stomachache, and other physical complications, consistent with the findings of a meta-analytic analysis report [88].

However, unlike most of the others’ study findings of WSH’s consequences in the hospitality workplaces, the reproductive health-related effects other than transactional sex practice such as engagement in commercial sex work, menstrual disorders, and acquiring STIs, including HIV, were reported in this study. The menstrual disorder issue as an effect of WSH is consistent with a quantitative study finding among female Italian university students [96]. This is a novel finding that has received little attention in the WSH literature. The Italian study found that age, place of birth, being in a couple of relationships, or hormone therapy did not affect these links and that sexual abuse during one’s life, depression, or a particular gynaecological diagnosis did not affect. According to this research team, changes in ovarian hormone levels and neurotransmitters, activation of the hypothalamic-pituitary-adrenal axis, or increased sensitivity to its activity are all possibilities. The team also supports the hypothesis described in conjunction with the hypothesis that emphasises the stress effect on the neurotransmitters affected by menstrual disorders. Stress may also lead to increased sensitivity in the perception of menstrual symptoms [97] and the maximum effect of stress in increasing menstrual symptom perception’s sensitivity, which needs further research about the link. This study’s finding implies that the effects of WSH are multidimensional and need multidisciplinary interventions supported by policies (both organisational and governmental). Though the mental, behavioural, physical health and organisational effect relations with WSH have been examined, studies did not show the relationship between WSH’s reproductive health effects, such as transactional sex practice and menstrual disorder, with other effects risks of WSH. Therefore, hospitality organisations should help WSH victims by providing psychological counselling, detaching themselves from workplace pressure, and effectively treating work-related depression. Legal protection and prevention measures (i.e., leadership engagement, solid legal protections, judicial protection services, WSH victims’ education, potential perpetrators, and the more comprehensive hospitality workplace staff about local laws; putting place prevention measures at the individual, community, and societal levels; organising workshops, training, and information sessions on how to bolster the resilience of WSH; and engagement all the stakeholders involved in the hospitality workplaces.), training for employees and managers, and financial support for women employees would also reduce vulnerability to WSH. Female hospitality workers’ protection from WSH should also need integrated legal security, well-versed prevention programs, and reproductive health links into legal frameworks. Future studies should also empirically test these relationships. A structural equation model incorporating the direct and indirect effects of the WSH on the identified consequences, which in turn helps in the understanding of moderators of the relationship between sexually harassing behaviours and the effects identified in this study, and multivariate analysis of variance incorporating all effects could be promising approaches. Exploring women employees’ WSH coping strategies could also give a complete picture.

While this study substantially contributes to the international academic literature on sexual harassment in the workplace, some limitations should be recognised. First, this study was conducted in Bahir Dar city, Amhara Region. It may not reflect WSH experiences in Ethiopia’s hospitality workplaces. Second, sexual harassment is a sensitive topic to discuss with different stakeholders (owners, supervisors, customers, cashiers, and women). Therefore, they may have under-reported such experiences (social desirability bias). However, the research team was made by public health professionals, health education, and behavioural science professionals trained to explore this multifaceted topic.

Furthermore, the first author was a man who led a team of female researchers to interview women about their workplace sexual harassment experiences, which mainly originated from male clients, and which necessitated the need to establish an atmosphere in which women felt secure sharing their actual experiences of harassment. Therefore, to maintain privacy, anonymity, and confidentiality of data, the first author explained to each team member and respondents that their identity and the evidence they would provide would be secret. Further, the first author and the team members were made clear to the respondents that only the researchers directly involved with this study would access it.

### Implications

This study adds essential insight into the existing research body on hospitality workplace sexual harassment in Ethiopia. The themes acknowledged here signify current social dynamics functioning within the hospitality workplaces’ setting and women relationships with customers, co-workers, agents, and immediate bosses. The behaviours and perceptions discussed suggest actionable areas for improving hospitality workplaces’ efforts to support women employees and prevent WSH through interventions. By providing female employees with practical support and security and promoting gender-equitable attitudes among employees, customers, co-workers, and immediate bosses, the hospitality workplaces can be a stepping-stone rather than a hurdle toward females’ realisation of their full potential. Likewise, organisational WSH related policies and strategies should have to be developed and implemented. The team also suggests that hospitality workplaces should formulate, in consultation with Ethiopian law and labour relations experts, to improve WSH policies that could change deep-rooted beliefs and norms. To the best of our knowledge, this is the first qualitative research in Ethiopia to investigate WSH perceptions, experiences, risks, and effects, especially reproductive health effects. However, the magnitude, associated factors, and consequences of the practice of WSH among women in hospitality workplaces should be a focus of future researches. Further research is also needed to document the coping and perpetrators’ personality.

## Conclusions

As suggested in the study findings, WSH is common. There remains a wide diversity of opinions regarding the meaning of WSH. Similarly, women employees faced various forms of WSH, mentioned different perceived risk factors and consequences. This finding indicates that WSH is a norm that creates a culture of hesitation and other unintended outcomes, which may endanger more health and organisations’ performance that seeks health care and prohibit productivity. Thus, it may cause a further rise in women’s psychological deterioration as long as social, moral, and legal norms are not harmonised. As a result, coordinated efforts from the hospitality industry, non-governmental organisations, government agencies, other stakeholders, sexual and reproductive health organisations, consumers, and female employees are needed.

## Supplementary Information

**Additional file 1.** Focus Group Guide for women hospitality workplace workers

**Additional file 2.** In-depth interview guide for women hospitality workplace workers

**Additional file 3.** In-depth interview guide for hospitality workplace managers

**Additional file 4.** In-depth interview guide for hospitality workplace customers

**Additional file 5.** In-depth interview guide for hospitality workplace cashiers

## Data Availability

The datasets used and analysed during the current study available from the corresponding author on reasonable request.

## Abbreviations

AIDS: Acquired Immune Deficiency Syndrome
CIRHT: Center for International Reproductive Health Training
FGDs: Focus Group Discussions
HIV: Human Immune Deficiency Virus
IDIs: In-depth Interviews
IRB: Institutional Review Board
KIIs: Key informant Interviews
PhD: Doctor of Philosophy
STIs: Sexually Transmitted Infections
USA: United States of America
WSH: Workplace Sexual Harassment
