# Supplementary material for: Perception and experiences of sexual harassment among women working in hospitality workplaces of Bahir Dar city, Northwest Ethiopia: a qualitative study
Source: BMC Public Health. 2021 Jun 11;21:1119. doi: 10.1186/s12889-021-11173-1 (PMC8196489; doi:10.1186/s12889-021-11173-1)
Supplement: Supplementary file 1 — Additional file 1. Focus Group Guide for women hospitality workplace workers [file 12889_2021_11173_MOESM1_ESM.docx]

**Focus Group Guide** **for women hospitality workplace workers**

**Consent form**

With the due understanding of the information above, are you willing to participate in the study? Yes

I, the undersigned, have been informed and understood that the purpose of this particular research project. I have been told that the information I give will be used only for this study; my identity, the information I provide will be treated confidentially. I have also been informed that I can refuse to participate in the study, not to respond to the question If I am not interested or stop responding to the interview at any time in the process. Based on the above information, I agree to participate in the research voluntarily.

Signature/finger print of the participants

1. Signature/finger print ________________ date _______________

2. Signature/finger print ________________ date _______________

3. Signature/finger print ________________ date _______________

4. Signature/finger print ________________ date _______________

5. Signature/finger print ________________ date _______________

6. Signature/finger print ________________ date _______________.

7. Signature/finger print ________________ date _______________

8. Signature/finger print ________________ date _______________.

9. Signature/finger print ________________ date _______________

10. Signature/finger print ________________ date _______________

1. Proceed with the interview
2. Terminate the interview

Name of moderator _____________________Signature: __________________­­­­­­­­­­­­­­­­­

Name of note taker _____________________signature: ___________________

Name of coordinator ________________________Signature: __________________

Date: ____________________________________

Note: In case of any unclarity you can communicate the principal investigator through the telephone Number: +251-913-288-238.

**Part I: General Information**

1. Date of the interview**:**/____/______/________/
2. Audio file no: _______________________ code no___________________
3. Venue: Blue Nile Hotel­­
4. Number of the participants: ___________________________
5. Category of the interview: Women working in hospitality workplaces

Part II. Respondents Background

(Do not write their name, code as P1, p2, p3…. p12 under the column headed P. code)

| NO | P. code | Age | Educational status | Roles | Birth place | Kebele |
| --- | --- | --- | --- | --- | --- | --- |
|  |  |  |  |  |  |  |
|  |  |  |  |  |  |  |
|  |  |  |  |  |  |  |
|  |  |  |  |  |  |  |
|  |  |  |  |  |  |  |
|  |  |  |  |  |  |  |
|  |  |  |  |  |  |  |
|  |  |  |  |  |  |  |

| No | Questions | Remark |
| --- | --- | --- |
| 1. 101 | How do you understand sexual harassment? |  |
| 1. 102 | Can you tell us about any related acts of sexual violence to the women working in Hospitality workplaces? Probes: Sexual jokes, Attempted to draw you into sexual banter, Made offensive sexual remarks in public or private, Attempted to make a romantic relationship with you, Treated you differently because of your sex, Made you feel like you were being bribed with rewards or consequences, Touched in an uncomfortable way |  |
| 1. 103 | How frequently acts of sexual harassment against women working in hospitality workplaces occur in your working place? |  |
| 1. 104 | Who are the potential perpetrators of sexual harassment the women working in hospitality workplaces?  Probe: customer, supervisor manager, co-worker |  |
| 1. 105 | Where do you go when you seek help concerning sexual harassment perpetration? |  |
| 1. 106 | What do you think is the cause of sexual harassment against women working in hospitality workplaces? **Probe:** Behavior, Clothing, |  |
| 1. 107 | What do you think is/are the impact of sexual harassment against women working in hospitality workplaces? Probe: Sexually transmitted disease, Mental health, Suicidal behavior, Social ostracisation. |  |
| 1. 108 | Anything else you would like to add? |  |

| 1. 108 | What have been/are the practice and trends of responding to sexual harassment against women working in hospitality workplaces?  Probe:   - What are the barriers that you face during your responses? - What deter you from responding to sexual harassment that you experience? |  |
| --- | --- | --- |
| 1. 109 | Are there opportunities for working with law enforcement agents and health workers to ensure the collaborative and integrated approach against the sexual harassment of women working in hospitality workplaces? |  |
| 1. 110 | What is your assessment of existing laws on sexual harassment against women working in hospitality workplaces? |  |
| 1. 111 | What do you think are the possible solutions for sexual harassment against women working in hospitality workplaces?  Probe: Training for women working in hospitality workplaces? Formulate and implement rules and regulations, awareness of customers about sexual harassment, how can this be improved upon? |  |
| 1. 112 | Anything else you would like to add? |  |
