# Supplementary material for: Perception and experiences of sexual harassment among women working in hospitality workplaces of Bahir Dar city, Northwest Ethiopia: a qualitative study
Source: BMC Public Health. 2021 Jun 11;21:1119. doi: 10.1186/s12889-021-11173-1 (PMC8196489; doi:10.1186/s12889-021-11173-1)
Supplement: Supplementary file 2 — Additional file 2. In-depth interview guide for women hospitality workplace workers [file 12889_2021_11173_MOESM2_ESM.docx]

**In-depth interview guide for women hospitality workplace workers**

**Part II– Information sheet**

**Introduction:**

Good morning/afternoon! My name is _________________. I represent the research team from Jimma University. We have chosen to conduct a study on sexual harassment in the hospitality industry. We are looking for participants to answer in-depth interview questions regarding sexual harassment, give comments on sexual harassment, and provide general demographic information. We request you to take part in our study. Your participation is voluntary, your identity remains confidential to me, and you may withdraw from the study at any time. You can correct any quotes made by checking the notes and transcripts from your interview so you can give feedback if you wish and withdraw anything you are not happy with.

**Purpose of the research**

This research aims to take an in-depth look at social trends in sexual harassment in the hospitality industry. We aim to find out if sexual harassment can be prevented entirely in hospitality workplaces, and if so, what methods organizations can use to coach staff in techniques to control customer harassment.

**Procedures**

You are among the 12 key-informants selected purposively for this in-depth interview. I will ask you a few questions concerning sexual harassment in the hospitality workplaces in this community. The discussion will take about 1:00 hour. So, I request your volunteer participation.

**Risks and discomfort**

The interview may take some of your time. However, we try to make it short and to the point and guide you through the discussion.

**Benefits**

There is no direct profit to you, but based on the information you provide us, we will design strategies to improve the interventions targeted to strengthen workplace sexual harassment prevention to reduce the impacts of sexual harassment.

**Confidentiality**

The information that we collect in this study will be kept confidential. Your name will not be written in this form, and the information we collect from you will not be publicized to anyone outside of this research. The collected data will also be used in aggregated form. The hard copies will also be kept in a locked cabinet and will not be divulged to anyone except the investigators.

**Right to refuse or withdraw**

You can refuse to answer any question to which you are not comfortable. You may stop participating in the interview at any time if not convenient for you without losing any of your rights as a participant. However, your active participation and genuine responses have paramount importance in improving neonatal health services in the future.

**Certificate of consent**

With the due understanding of the information above, are you willing to participate in the study? Yes

I have been requested to take part in the research, and the preliminary report has been read to me. I have had the chance to ask questions about it, and any questions I have been requested have been answered to my satisfaction. I consent voluntarily to participate in this study and understand that I have the right to withdraw from the interview at any time without in any way affecting my right.

**Signature/fingerprint of the participant**

Signature/fingerprint ________________ date _______________

1. Proceed with the interview
2. Terminate the interview

Name of moderator _____________________Signature: __________________­­­­­­­­­­­­­­­­­

Name of note-taker _____________________signature: ___________________

Name of coordinator ________________________Signature: __________________

Date: ____________________________________

Note: In case of any unclarity, you can communicate the principal investigator through the telephone Number: +251-913-288-238.

**Part I: General information**

101: Date of the interview**: /**____/______/________/

102: Code no/___________________/

103: Kebele: /__________________________________/

104: Category of the interview**: -**women working in hospitality workplaces

| **Part II: Socio-Demographic Characteristics Respondents** | | | |
| --- | --- | --- | --- |
| NO | **Questions** | **Remark** | |
| 201 | Age: /_______________/ complete year |  | |
| 202 | Profession:/ ______________________________________________________/ |  | |
| 203 | Educational status: /________________________________________________/ |  | |
| 204 | Position held: /waiter/ |  | |
| 205 | Service year:/__________________________________________/ |  | |
| 206 | **Opening questions:**   1. What is your work experience as a woman working in hospitality workplaces? |  | |
| **Part III: Sexual Harassment Related Questions** | | | |
| 207 | How do you understand sexual harassment?  How do you understand the characteristics of sexual harassment? | |  |
| 208 | Tell me about any incidents where you were made non-comfortable, or treated non-appropriately.  Probe: What exactly happened? What correctly did he do/say? Verbal? How explicit? Physical? What context did it occur? Over what period did it occur? Frequency? Was anyone else aware of the behavior? Any other incidents? | |  |
| 209 | - What was your relationship with the sexual harasser both before and during the sexual harassment? Who harassed you? How much is the influence of the harasser on this facility? Did the harasser have the power to take the business elsewhere? - How much of your personal life is reliant on the harasser? If you lost your job, would you suffer? | |  |
| 210 | - What is your opinion on sexual harassment in the hospitality industry? - Do you consider it to be a problem within the industry? - Have you ever been touched with the issue? How? | |  |
| 211 | How do women who work in hospitality workplaces and targeted for sexual harassment respond to sexual harassment that they experienced in the short and long term? | |  |
| 212 | What do you think are the possible solutions for the prevention of sexual harassment against women working in hospitality workplaces?  How do women who are the victims of sexual harassment, understand their experience to improve their careers?  What strategies for preventing and responding to sexual harassment in hotels, restaurants, beverage groceries, and cafeterias perceive as promising?  What are the barriers and challenges you believe are present to prevent sexual harassment in hotels, restaurants, beverage groceries, and cafeterias from being addressed?  Probe: Provide training for women working in hospitality workplaces, Formulation, and implementation of rules and regulations, creation of awareness of customers about sexual harassment. | |  |
| 213 | Is there anything else you would like to add? | |  |
