# Supplementary material for: Perception and experiences of sexual harassment among women working in hospitality workplaces of Bahir Dar city, Northwest Ethiopia: a qualitative study
Source: BMC Public Health. 2021 Jun 11;21:1119. doi: 10.1186/s12889-021-11173-1 (PMC8196489; doi:10.1186/s12889-021-11173-1)
Supplement: Supplementary file 3 — Additional file 3. In-depth interview guide for hospitality workplace managers [file 12889_2021_11173_MOESM3_ESM.docx]

**In-depth interview guide for hospitality workplace managers**

- Name of moderator _____________________Signature: __________________­­­­­­­­­­­­­­­­­
- Name of note taker _____________________signature: ___________________
- Name of coordinator ________________________Signature: __________________
- Date: ____________________________________

**Part I: General information**

101: Date of the interview**: /**____/______/________/

102: Code no/___________________/

103: Kebele: /__________________________________/

104: Category of the interview**: /Manager/**

| **Part II: Socio-Demographic Characteristics Respondents** | | | |
| --- | --- | --- | --- |
| NO | **Questions** | **Remark** | |
| 201 | Age: /_______________/ complete year |  | |
| 202 | Sex: Male /_______/ Female /_______/ |  | |
| 203 | Profession: / ______________________________________________________/ |  | |
| 204 | Educational status: /________________________________________________/ |  | |
| 205 | Position held: /Manager/ |  | |
| 206 | Service year: /__________________________________________/ |  | |
| 207 | Can you briefly tell me about yourself and your role at the hotel? What is your experience as a manager? |  | |
| 208 | Can you briefly tell me about the hotel and the structure of the work? How many employees do you have? How does the organization look? |  | |
| **Part III: Sexual harassment Related Questions** | | | |
| 301 | What are some of the difficulty’s the women who work in hospitality workplaces have known to face in hotels, restaurants, and cafeterias? Probe: unwanted sexual attention, gender harassment, sexual coercion | |  |
| 302 | By whom do you think to commit most of the sexual harassment against women working in hospitality workplaces? Probe: Customer, Manager, Peer, Broker, The Ex-boyfriend | |  |
| 303 | Why do you think is the possible reason that women who are working in hospitality workplaces are targeted to harass sexually? Probe: Women who are working in hospitality workplaces behavior/interest, customer's perception, pressure from the manager…. | |  |
| 304 | How do the women who are working in hospitality workplaces respond to sexual harassment (action)? Ignore the behavior? Confront the harasser? Go along with it / tolerate it to avoid confrontation? Attempt to prevent the harasser? In what ways? Report the practice to anyone? To whom? Did you think your organization would take waitresses complaints’ seriously? | |  |
| 305 | What do you think is the outcome of being a victim of sexual harassment? Profession: Relationship with a client changed, Lost a client’s business? Got reassigned? Absenteeism? Less motivated at work? “Withdrawal” Behaviors. Personal: Depression, Self-esteem, Illness | |  |
| 306 | What is your opinion on sexual harassment in the hospitality industry? Do you consider it to be a problem within the industry? Have you ever been in touch with the issue? How? | |  |
| 307 | Is there a strategy in how to work with SH within the company? In this workplace? Do you have a routine action that you follow in case women who are working in hospitality workplaces have faced sexual harassment in this place? If so, how does the strategy look? With whom did you implement the plan to address the women who are working in hospitality workplaces sexual harassment? Do you have any real encounter with the waitress’s sexual harassment in this facility? What happened? How did you react? What do you consider to be your responsibility as hotel managers to work with this issue? What precautions can you take to work against the problem in your facility? | |  |
| 308 | What do you think are the possible solutions to prevent sexual harassment against women who are working within hospitality workplaces? Probe: Implement training for women who are working in hospitality workplaces, Formulate and implement rules and regulations, creation of awareness of customers about sexual harassment | |  |
| 309 | Anything else you would like to add? | |  |
