# Supplementary material for: Perception and experiences of sexual harassment among women working in hospitality workplaces of Bahir Dar city, Northwest Ethiopia: a qualitative study
Source: BMC Public Health. 2021 Jun 11;21:1119. doi: 10.1186/s12889-021-11173-1 (PMC8196489; doi:10.1186/s12889-021-11173-1)
Supplement: Supplementary file 4 — Additional file 4. In-depth interview guide for hospitality workplace customers [file 12889_2021_11173_MOESM4_ESM.docx]

**In-depth interview guide for hospitality workplace customers**

Name of moderator _____________________Signature: __________________­­­­­­­­­­­­­­­­­

Name of note taker _____________________signature: ___________________

Name of coordinator ________________________Signature: __________________

Date: ____________________________________

Part I: General information

101: Date of the interview**: /**____/______/________/

102: Code no/___________________/

103: Kebele: /__________________________________/

104: Category of the interview**: Customer**

| **Part II: Background of the Respondents** | | | |
| --- | --- | --- | --- |
| NO | **Questions** | **Remark** | |
| 201 | Age: /_______________/ complete year |  | |
| 202 | Profession: / ______________________________________________________/ |  | |
| 203 | Educational status: /________________________________________________/ |  | |
| 204 | **The questions for the opening:**   - How long have you been a customer? |  | |
| **Part III: Sexual Harassment Related Questions** | | | |
| 301 | - What are some of the difficulty’s the women who work in hospitality workplaces have known to face in hotels, restaurants, and cafeterias? Probe: unwanted sexual attention, gender harassment, sexual coercion | |  |
| 302 | - By whom do you think to commit most of the sexual harassment against women working in hospitality workplaces? Probe: Customer, Manager, Peer, Broker, The Ex-boyfriend | |  |
| 303 | - Why do you think is the possible reason that women who are working in hospitality workplaces are targeted to harass sexually? Probe: When working in hospitality workplaces behavior/interest, customer's perception, pressure from the manager…. | |  |
| 304 | - What do you think is the outcome of being a victim of sexual harassment? Profession: Relationship with a client changed, Lost a client’s business? Got reassigned? Absenteeism? Less motivated at work? “Withdrawal” Behaviors. Personal: Depression, Self-esteem, Illness | |  |
| 305 | - What is your opinion on sexual harassment in the hospitality industry? Do you consider it to be a problem within the industry? Have you ever been touched with the issue? How? | |  |
| 306 | - What do you think are the possible solutions to prevent sexual harassment against women who are working within hospitality workplaces? Probe: Implement training for women who are working in hospitality workplaces, Formulate and implement rules and regulations, creation of awareness of customers about sexual harassment | |  |
| 307 | Anything else you would like to add? | |  |
