# Supplementary material for: Perception and experiences of sexual harassment among women working in hospitality workplaces of Bahir Dar city, Northwest Ethiopia: a qualitative study
Source: BMC Public Health. 2021 Jun 11;21:1119. doi: 10.1186/s12889-021-11173-1 (PMC8196489; doi:10.1186/s12889-021-11173-1)
Supplement: Supplementary file 5 — Additional file 5. In-depth interview guide for hospitality workplace cashiers [file 12889_2021_11173_MOESM5_ESM.docx]

**In-depth interview guide for hospitality workplace cashiers**

Name of moderator _____________________Signature: __________________­­­­­­­­­­­­­­­­­

Name of note taker _____________________signature: ___________________

Name of coordinator ________________________Signature: __________________

Date: ____________________________________

Part I: General information

101: Date of the interview**: /**____/______/________/

102: Code no/___________________/

103: Kebele: /__________________________________/

104: Category of the interview**: /cashier/**

| **Part II: The socio-demographic characteristics of the Participants** | | | |
| --- | --- | --- | --- |
| NO | **Questions** | **Remark** | |
| 201 | Age: /_______________/ complete year |  | |
| 202 | Sex: Male /_______/ Female /_______/ |  | |
| 203 | Profession: / ______________________________________________________/ |  | |
| 204 | Educational status: /________________________________________________/ |  | |
| 205 | Position held: /__________________________________________________/ |  | |
| 206 | Service year: /__________________________________________/ |  | |
| 207 | Can you briefly tell me about yourself and your role at the hotel? What is your work experience as a cashier? How long did you work as a cashier? |  | |
| **Part III: Questions Related to Sexual harassment** | | | |
| 301 | - What are some of the difficulty’s the women who work in hospitality workplaces have known to face in hotels, restaurants, and cafeterias? Probe: unwanted sexual attention, gender harassment, sexual coercion | |  |
| 302 | By whom do you think to commit most of the sexual harassment against women working in hospitality workplaces? Probe: Customer, Manager, Peer, Broker, The Ex-boyfriend | |  |
| 303 | Why do you think is the possible reason that women who are working in hospitality workplaces are targeted to harass sexually? Probe: Women working in hospitality workplaces behavior/interest, customer’s perception, pressure from the manager…. | |  |
| 304 | - How do women who work in hospitality workplaces and are targeted for sexual harassment respond to sexual harassment that they experienced in the short term? Did you think your organization would take waitresses complaints’ seriously? | |  |
| 305 | - What do you think is the outcome of being a victim of sexual harassment? Profession: Relationship with a client changed, Lost a client’s business? Got reassigned? Absenteeism? Less motivated at work? “Withdrawal” Behaviors. Personal: Depression, Self-esteem, Illness | |  |
| 306 | 1. What is your opinion on sexual harassment in the hospitality industry? Do you consider it to be a problem within the industry? Have you ever been in touch with the issue? How? | |  |
| 307 | 1. What do you think are the possible solutions to prevent sexual harassment against women who are working within hospitality workplaces? Probe: Implement training for women who are working in hospitality workplaces, Formulate and implement rules and regulations, creation of awareness of customers about sexual harassment | |  |
| 309 | Anything else you would like to add? | |  |
